# The Role of Cellular Prion Protein in Promoting Stemness and Differentiation in Cancer

**DOI:** 10.3390/cancers13020170

**Published:** 2021-01-06

**Authors:** Larisa Ryskalin, Francesca Biagioni, Carla L. Busceti, Maria A. Giambelluca, Luca Morelli, Alessandro Frati, Francesco Fornai

**Affiliations:** 1 Department of Translational Research and New Technologies in Medicine and Surgery, University of Pisa, Via Roma 55, 56126 Pisa, Italy; larisa.ryskalin@unipi.it (L.R.); maria.giambelluca@unipi.it (M.A.G.); 2 Istituto di Ricovero e Cura a Carattere Scientifico (I.R.C.C.S.) Neuromed, Via Atinense 18, 86077 Pozzilli, Italy; francesca.biagioni@neuromed.it (F.B.); carla.busceti@neuromed.it (C.L.B.); alessandro.frati@uniroma1.it (A.F.); 3 General Surgery Unit, Department of Translational Research and New Technologies in Medicine and Surgery, University of Pisa, 56124 Pisa, Italy; luca.morelli@unipi.it; 4 EndoCAS (Center for Computer Assisted Surgery), University of Pisa, 56124 Pisa, Italy; 5 Neurosurgery Division, Human Neurosciences Department, Sapienza University, 00135 Roma, Italy

**Keywords:** cellular prion protein, cancer stem cells, brain tumors, peripheral tumors, tumorigenesis, self-renewal, differentiation

## Abstract

**Simple Summary:**

Aside from its well-established role in prion disorders, in the last decades the significance of cellular prion protein (PrP^C^) expression in human cancers has attracted great attention. An extensive body of work provided evidence that PrP^C^ contributes to tumorigenesis by regulating tumor growth, differentiation, and resistance to conventional therapies. In particular, PrP^C^ over-expression has been related to the acquisition of a malignant phenotype of cancer stem cells (CSCs) in a variety of solid tumors, encompassing pancreatic ductal adenocarcinoma, osteosarcoma, breast, gastric, and colorectal cancers, and primary brain tumors as well. According to consensus, increased levels of PrP^C^ endow CSCs with self-renewal, proliferative, migratory, and invasive capacities, along with increased resistance to anti-cancer agents. In addition, increasing evidence demonstrates that PrPc also participates in multi-protein complexes to modulate the oncogenic properties of CSCs, thus sustaining tumorigenesis. Therefore, strategies aimed at targeting PrP^C^ and/or PrP^C^-organized complexes could be a promising approach for anti-cancer therapy.

**Abstract:**

Cellular prion protein (PrP^C^) is seminal to modulate a variety of baseline cell functions to grant homeostasis. The classic role of such a protein was defined as a chaperone-like molecule being able to rescue cell survival. Nonetheless, PrP^C^ also represents the precursor of the deleterious misfolded variant known as scrapie prion protein (PrP^Sc^). This variant is detrimental in a variety of prion disorders. This multi-faceted role of PrP is greatly increased by recent findings showing how PrP^C^ in its folded conformation may foster tumor progression by acting at multiple levels. The present review focuses on such a cancer-promoting effect. The manuscript analyzes recent findings on the occurrence of PrP^C^ in various cancers and discusses the multiple effects, which sustain cancer progression. Within this frame, the effects of PrP^C^ on stemness and differentiation are discussed. A special emphasis is provided on the spreading of PrP^C^ and the epigenetic effects, which are induced in neighboring cells to activate cancer-related genes. These detrimental effects are further discussed in relation to the aberrancy of its physiological and beneficial role on cell homeostasis. A specific paragraph is dedicated to the role of PrP^C^ beyond its effects in the biology of cancer to represent a potential biomarker in the follow up of patients following surgical resection.

## 1. Background

Prion protein is renowned for its causative role in the pathogenesis and transmission of prion diseases (Prusiner, 1998) [1]. Also known as transmissible spongiform encephalopathies (TSEs), prion diseases are progressive, irreversible, and fatal neurodegenerative disorders, affecting both humans and other mammals.

In humans, TSEs include Creutzfeldt-Jakob disease (CJD), Gerstmann-Sträussler-Scheinker disease (GSS), fatal familial insomnia (FFI), kuru, and most recently variant of CJD (vCJD), whereas in animals TSEs comprise bovine spongiform encephalopathy (BSE) in cattle, scrapie in sheep and goats, and chronic wasting disease (CWD) in cervids [1]. Being either sporadic, inherited, or infectious [2], these disorders are characterized by spongiform degeneration of the central nervous system (CNS) [3], neuronal loss, and astrogliosis [4]. Independently from their origin, the neuropathological hallmark of prion disorders is the accumulation of protein aggregates within the brain containing a deleterious and transmittable isoform of the normal PrP, named PrP scrapie (PrP^Sc^) [4]. PrP^Sc^ represents an altered and misfolded isoform of the cellular PrP (PrP^C^), which is normally expressed in eukaryotic cells [5,6]. Despite owning the same primary amino-acid sequence, PrP^Sc^ differs from the naturally occurring PrP^C^ in its secondary structure. Compared with PrPc, PrP^Sc^ is mainly characterized by large β-sheets, which become predominant over the α-helical and coil structures [7]. Notably, the reduction in α-helices, which are refolded into β-sheets, results in profound changes in the physio-chemical properties of PrP. In fact, while PrPc is soluble and highly susceptible to protease digestion, PrP^Sc^ is insoluble and protease-resistant, thus forming protein aggregates that precipitate within the cell. This is consistent with the observation that β-sheet-enriched amyloid deposits of PrP^Sc^ are abundant and accumulate within prion-infected brains [2,8,9]. Furthermore, the disease-associated PrP^Sc^ can translate other normal PrP^C^ into the pathological PrP isoform, acting as a seed that initiates protein refolding of a nascent PrP^C^ molecule [10]. This, in turn, fosters intra-/extra-cellular accumulation of insoluble protein aggregates, which ultimately results in cellular dysfunction and neurotoxicity [11]. Again, any dysfunction in PrP removal increases the probability of spontaneous PrP^Sc^ generation. Although prion metabolism is partly dependent on the ubiquitin proteasome system, the clearance of PrP^Sc^ is tightly bound to the activity of the main protein degradation pathway, namely autophagy (ATG) [12,13,14]. When a high amount of PrP^C^ occurs, this protein cannot be promptly degraded and its accumulation above a certain threshold over time enables PrP misfolding and aggregation, while clogging cell-clearing pathways [15]. Thus, when ATG is overwhelmed, PrP further accumulates within the cell, thereby exacerbating PrP pathogenic conversion, self-propagation, and spreading. As a proof of concept, ATG-inducing agents enhance misfolded PrP degradation thus preventing its aggregation into amyloids [12,16,17,18]. On the other hand, the clearance of aggregate-prone proteins is impaired when ATG is suppressed, either pharmacologically by known ATG inhibitors or genetically by siRNA targeting ATG genes [13,16].

Notwithstanding the recognition of the infectious potential of PrP, its significance extends way beyond prion diseases. In the last decades, PrP^C^ has gathered great attention for its involvement in tumor cell biology. A growing body of evidence indicates that PrPc over-expression contributes to brain tumorigenesis by regulating tumor growth, invasiveness, and therapeutic resistance. Remarkably, PrP^C^ over-expression occurs in various tumors of the nervous system, encompassing meningioma, medulloblastoma, schwannoma, and glioma, mostly glioblastoma multiforme (GBM) [19,20,21,22,23]. Among primary brain tumors, GBM represents the highest, most aggressive, and severe prognostic grade (WHO grade IV glioma). Intriguingly, increased PrP^C^ expression in human glioma samples correlates with tumor grade and thus lower patients’ overall survival. In fact, human GBM samples feature higher levels of PrP^C^ than low-grade glioma (LGG, grades I–II) and grade III astrocytoma [20,23].

It is worth mentioning that several lines of evidence indicate that PrP^C^ plays a role not only in the nervous system but also throughout the human body. In fact, although being highly expressed within the CNS, PrP^C^ also occurs in various human peripheral tissue and organs. Remarkably, the discovery of PrP^C^ expression in different cell types joined the evidence of PrP^C^ over-expression in various human cancers [24]. As recently reported, PrP^C^ is highly expressed in a variety of solid tumors, including gastric and colorectal cancer [25,26,27,28,29], breast cancer [30,31,32], prostate cancer [33], pancreatic ductal adenocarcinoma (PDAC) [34,35,36], lung adenocarcinoma [37], head and neck squamous cell carcinoma (HNSCC) [38], osteosarcoma [39], and melanoma [40,41]. Again, PrP^C^ over-expression is closely associated with tumor malignancy and poor prognosis [42,43,44,45].

Looking towards the comprehension of the role of PrP^C^ in the biology of cancer, consistent evidence suggests that PrP^C^ is involved in the proliferation, migration, invasion, and therapeutic resistance of cancer stem cells (CSCs). Regardless of the cell of origin, this applies to both hematopoietic and solid tumors. Virtually present in any kind of tumor, CSCs represent a small subset of cancer cells endowed with key features of normal stem cells, such as sustained self-renewal and proliferation [46]. Thus, CSCs are thought to be the driving force of tumorigenesis since they can initiate and sustain tumor growth and progression. Moreover, CSCs possess an enhanced disseminating capacity and increased therapeutic resistance enabling these cells to invade neighboring healthy tissues and/or metastasize to distant organs [46]. Consistent evidence indicates that PrPc over-expression is key to sustain CSC self-renewal, clonogenicity, and tumorigenic potential [35,47,48,49]. Conversely, its inhibition and/or down-regulation results in a more differentiated, less oncogenic CSC phenotype [22,29,50,51]. Again, PrPc down-regulation restores CSC sensitivity to chemo- and radiotherapy [52].

It is also noteworthy that, beyond its effects in cancer biology, emerging evidence suggests that PrP^C^ expression may have a diagnostic value in various solid tumors [35,45,53]. Recently, PrP^C^ emerged as a potential biomarker in the follow-up of patients following surgical resection of PDAC [36]. Thus, PrP^C^ may become a useful tool in monitoring the therapeutic efficacy as well as predicting the outcome of cancer patients undergoing chemo- and radiotherapy [34,44,54,55,56].

Therefore, in the next paragraphs we provide evidence regarding the physiological and beneficial role of PrP^C^ on cell homeostasis. Then we move forward to discuss recent data regarding the multiple effects of PrP^C^ in sustaining cancer initiation, progression, and recurrence, with a special focus on the role of PrP^C^ in CSC biology. These aspects, which are seminal in cancer research, may provide novel insights on the role of PrP^C^ as both a prognostic biomarker and a potential therapeutic target to force CSC to shift towards more differentiated and therapy-sensitive cancer cells. The findings herein discussed may contribute to early diagnosis of cancer, while potentially improving future targeted interventions.

## 2. The Physiological Role of Cellular Prion Protein (PrP^C^)

### 2.1. Structure, Biogenesis, and Intracellular Trafficking of PrP^C^

The normal cellular prion protein (PrP^C^) is an endogenous, highly conserved cell-surface glycoprotein encoded by the PRNP gene [57]. In humans, PRNP transcripts are detected at a variable extent in various peripheral tissues (e.g., gastrointestinal tract, lung, heart, mammary glands), and to a higher level within the central and peripheral nervous system [58]. In fact, PrPc is most abundant within neurons and glia of selective brain areas, although they are also quite ubiquitously distributed in non-neuronal cell types [4].

The newly synthesized PrP^C^ is a 253 amino acid polypeptide composed of an unstructured N-terminal domain and a globular C-terminal domain, which contains three α-helices and two β-sheets [59]. In particular, the N-terminal signal peptide is essential for the translocation of immature PrP^C^ into the lumen of the endoplasmic reticulum (ER). However, some PrP^C^ molecules fail to properly translocate into the ER, and thus, they are retained within the cytosol [60].

The biogenesis of PrP^C^ requires a series of co- and post-translational modifications. In particular, the nascent protein enters into the ER lumen, where the N-terminal signal peptide is rapidly removed. Following the cleavage of the N-terminal flexible tail, PrP^C^ undergoes a second cleavage at the carboxy-terminal globular domain along with a glycosyl-phosphatidyl-inositol (GPI) modification at residue 230. In detail, the C-terminal GPI anchor peptide signal sequence (GPI-PSS) is removed, while being rapidly replaced with a GPI anchor that tethers PrP^C^ to the outer leaflet of the plasma membrane (PM) [61]. The removal of both signal peptides results in a mature PrP^C^, which consists of 208 amino acids. Then, the protein moves to the Golgi apparatus to undergo post-translational modifications [62]. In particular, PrP^C^ undergoes N-glycosylation at two highly conserved sites in its C-terminal, namely Asn-181 and Asn-197 residues [61]. These latter may be occupied by sugar moieties at a variable extent, thus resulting in different glycosylated forms of PrP^C^ (i.e., monoglycoylated, diglycosylated, and unglycosylated) [63]. This, in turn, stabilizes PrP^C^ secondary structure, while favoring its correct localization to the PM [61,64].

However, in different human cancer cell lines and tissues such as PDAC and melanoma, PrP is incompletely processed and exists as a precursor form of normal PrP^C^ [34,40,65]. This latter, known as pro-PrP, represents an alternative form of the mature, full-length PrP^C^. In particular, pro-PrP lacks the N-terminal signal peptide, the sugar moieties, and the GPI, while retaining the normally cleaved GPI-PSS [65]. Remarkably, the GPI-PSS contains several small hydrophobic amino acids, which in turn, are responsible for the unconventional insertion of pro-PrP into the phospholipid bilayer of the PM, rather than to the PM outer face, as occurring for mature PrP^C^.

The GPI-anchored PrP^C^ is strategically associated with lipid rafts, which implies that this protein is involved in signal transduction and cell-to-cell communication [66]. As well as its localization within cholesterol-rich lipid rafts, PrP^C^ is also internalized through caveolin-dependent endocytosis and/or clathrin-coated pits [67,68]. In particular, recent studies demonstrate that PrP^C^ moves towards the non-raft region of the PM to interact with the low-density lipoprotein receptor-related protein 1 (LPR1), thus undergoing receptor-mediated endocytosis [69]. Furthermore, increasing evidence in both neuronal and non-neuronal cell types demonstrates the association of PrP^C^ with exosomes [70,71,72,73,74]. These latter are small extracellular vesicles (EVs) which participate in protein homeostasis and contribute to the maintenance of cellular fitness [75]. In particular, exosomes represent the smallest EVs (30–100 nm in diameter) and they originate from the endosomal system upon fusion of multivesicular bodies (MVBs) with the PM. In fact, most cell types remove unwanted and/or damaged material in a constitutive manner through the release of exosomes. Remarkably, exosomal functions extend way beyond the removal of waste material within the extracellular space. In fact, given that exosomes carry several proteins and nucleic acids that can be transferred to neighboring cells, these nanovesicles recently emerged as an unconventional mechanism of cell-to-cell communication, in normal physiological conditions as well as in pathological progression [76,77].

### 2.2. The Physiological and Beneficial Role of PrP^C^

In attempts to unravel the putative functions of PrP^C^, in the early 1990s several PRNP knock-out (KO) mice models were generated. Even though these experimental models helped to demonstrate the infectious potential of PrP, its physiological role still remained enigmatic. In fact, whilst PrP^C^ KO prevented scrapie infection, these transgenic mice developed normally without any apparent CNS structural changes and/or behavioral alterations [78,79,80]. Similarly, pioneer studies in cattle and goats have indicated that PRNP KO animals do not show any developmental alterations or abnormal behavior when compared with controls [81,82]. However, as recently reported, the absence of a remarkable phenotype in these transgenic animals might be due to the compensatory role of two proteins belonging to the PrP family, namely Doppel and Shadoo [83,84]. Nonetheless, recent studies demonstrated that mice lacking PrP^C^ or expressing a mutant PrP^C^ isoform resulted in severe motor alterations due to impaired excitability and synaptic plasticity within cerebellar granule neurons [85,86].

Contrary to in vivo studies, in vitro models revealed a plethora of cellular functions that have been ascribed to PrP^C^. For instance, within the CNS, PrP^C^ is involved in neurite extension, neuronal differentiation, and neuroprotection [84,87,88]. Moreover, albeit promoting differentiation of tissue-resident stem cells, PrP^C^ may also promote stemness and cell proliferation, depending on specific conditions [84]. Intriguingly, recent studies pointed to a key role for PrP^C^ in the transcriptional regulation of pluripotency and stemness genes of hematopoietic, mammary gland, mesenchymal, embryonic, and neural stem cells, but also influences stem cell fate and cell cycle [89,90,91,92]. More in general, PrP^C^ is involved in copper metabolism, cell proliferation, adhesion, and migration [16].

Remarkably, most PrP^C^ biological functions appear to be linked to its binding partners [93]. Several lines of evidence are consistent with a role of PrP^C^ as a molecular scaffold protein involved in signal transduction. In fact, PrP^C^ activity depends on its specific localization within lipid rafts of the PM, where it interacts with a variety of receptors and molecules to transduce intracellular signals. Notably, recent investigations on PrP-interacting proteome (i.e., interactome) helped to disclose, at least in part, the elusive PrP^C^ biology [94,95,96]. In particular, PrP^C^ binds to several cell-surface components, such as the neural cell adhesion molecule (NCAM), LRP1, stress-inducible protein 1/Hsp70/Hsp90 organizing protein (STI1/HOP), 37-kDa/67-kDa laminin receptor precursor/laminin receptor (LPR/LR), filamin A (FLNa), and Notch 1 [35,97,98]. In this way, PrPc modulates the activity of various signaling pathways and/or signaling components including PI3K/Akt/mTOR, cAMP/PKA, MAPK/ERK, PKC, and Fyn kinase [87,99,100,101] For instance, PrP^C^ interacts with the NCAM to promote PrP-dependent neurite outgrowth through activation of the cytosolic Fyn kinase [88]. Likewise, the interaction between several extracellular matrix components (ECM) and transmembrane receptors (i.e., laminin, integrin β1, LRP1, and EGFR) can elicit PrP-mediated neurotrophic effects [95,102,103,104,105].

### 2.3. PrP^C^ Functions in Cell Survival and Stress Protection

Recent data relate the physiological role of PrP^C^ in promoting cell survival, while rescuing the cell under stressful conditions [30,106,107,108]. In fact, it has been reported that PrP^C^ exerts cytoprotective activity, particularly as regards protection against serum deprivation, DNA damage, and apoptotic and oxidative stress [93,106,109]. Furthermore, PrP^C^ specifically prevents pathological protein aggregation in neuronal cells. For instance, PrP^C^ confers neuroprotection against huntingtin (Htt)-induced toxicity, while its depletion contributes to a loss of function under stress conditions [110]. In fact, depletion of endogenous PrP^C^ by RNA interference (RNAi) in neuronal cell lines results in a marked increase in Htt aggregation, along with a significant reduction in antioxidant enzymes and proteasome activity [110]. Thus, PrP^C^ emerges as seminal in modulating a variety of baseline cell functions to grant cell homeostasis.

The classic role of such a protein was defined as a chaperone-like molecule being able to rescue cell survival. Notably, PrP^C^ associates with several molecular chaperones and co-chaperones such as the heat shock protein (Hsp60) [111], Hsp40 and Hsps70 [112,113], BiP [112], STI1/HOP [22,101], and αβ-crystallin [114]. In particular, these molecular chaperones exhibit a cytoprotective function since they promote proper protein folding and facilitate correct refolding, while disassembling protein aggregates [115,116]. This, in turn, is essential for preserving cellular homeostasis under physiological conditions, while being crucial under stressful conditions to guarantee cell survival.

Mapping of PrP^C^ peptide showed that this protein possesses a highly conserved binding site for Hsp60, a widely distributed and highly conserved molecular chaperone that mediates ATP-dependent folding of polypeptide chains [111,117]. Likewise, PrPc directly binds to αβ-crystallin, a member of small Hsp (sHsp) [114]. Moreover, STI1/HOP, a critical co-chaperone of the Hsp70/Hsp90 machinery [74,100] interacts with PrP^C^ at the PM to trigger neuroprotective signals, while rescuing cells from apoptosis [99,100,118].

Interestingly, experimental evidence demonstrates that heat shock in human NT-2 cells results in the simultaneous induction of both PrP^C^ and Hsp70 [119]. In particular, heat stress-induced PrP transcription and translation occurs through the interaction of heat-shock transcription factor (HSTF) on PRNP gene promoter, resulting in increased protein synthesis [119]. Furthermore, PrP^C^ can act as an antioxidant chaperone to promote neural stem cells (NSC) survival and stemness. Upon stressful conditions (i.e., serum deprivation), an increase in intracellular reactive oxygen species (ROS) levels promotes β-mediated proteolytic cleavage of PrP^C^ into a C-terminal and N-terminal fragment [120,121]. The latter is released extracellularly to trigger the MEK1 pathway, which sustains the antioxidant effects of PrP^C^ [122]. Thus, it is conceivable that PrP^C^ may act as an Hsp in protecting cells from the deleterious effects of heat, serum deprivation, and ROS.

## 3. The Multi-Faceted Role of PrP^C^ in Cancer initiation and Progression

**Figure 1:**
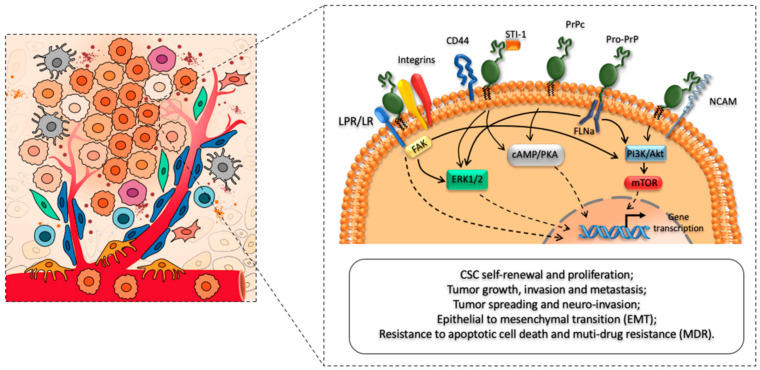
Cellular prion protein (PrP^C^) as a main regulator of CSCs phenotype, biology, and functioning. The left panel of the cartoon depicts the cross-talk between cancer stem cells (CSCs) and major cellular components (i.e., endothelial cells, fibroblasts, pericytes, macrophages, and T cells) of the tumor micro-environment (TME). The right panel schematizes the role of PrP^C^ in promoting CSC self-renewal, proliferation, and migration. In addition, PrP^C^ interacts with several receptors and cell surface proteins to modulate CSCs’ tumor-initiating and metastatic capacities, while promoting increased therapeutic resistance. In this way, it modulates tumor growth, survival, infiltration, and multi-drug resistance.

In addition to being implicated in the pathogenesis and transmission of prion diseases, increasing evidence demonstrates that PrP^C^ contributes to tumorigenesis via multiple pathways by regulating tumor growth, differentiation, and resistance to conventional therapies [21,22,23,28,31,35,47] (Figure 1). Such a role may not be restricted to primary brain tumors, since PrP^C^ is ubiquitously expressed throughout the human body. Given its widespread expression among different non-neuronal cell types, it is reasonable that PrP^C^ plays a key role in various tumors, beyond CNS neoplasms [25,29,32,33,36,37,38]. In fact, mounting evidence indicates that PrP^C^ is highly expressed in several cancer cell types and its over-expression is associated with increased tumor aggressiveness and invasiveness.

Over the past few years, several lines of evidence raised the possibility that PrP^C^ may act way beyond human embryonic and adult resident stem cells [123], thereby implicating PrPc in the biology of CSCs. These latter, which represent a fraction of tumor cells endowed with key feature of normal stem cells, have tumor-initiating potential and emerged as pivot to fuel tumor growth, infiltration, and relapse [124]. As a proof of concept, pioneer studies identified endogenous PrP^C^ to be highly expressed within different subpopulations of CSCs encompassing colorectal, gastric, lung, breast, and GBM cancer cell lines [28,30,37,45,48,125,126,127,128]. Aside from PRNP, other members of the prion gene family were found to be up-regulated in various tumor tissues and cancer cell lines. For instance, an over-expression of the prion-like protein gene PRND (Doppel, Dpl) at both protein and mRNA levels occurs in high-grade astrocytoma and GBM [19,20,129]. Similarly, high levels of PRND are found in non-glial malignant tumors such as gastric adenocarcinoma, anaplastic meningioma, and osteosarcoma [19,39].

Interestingly, a specific analysis of PRNP gene mutations using the Cancer Genome Atlas (TCGA) database revealed a total of 48 somatic mutations in the PRNP gene in different cancers, encompassing lung adenocarcinoma, colorectal adenocarcinoma, endometrial carcinoma, head and neck squamous cell carcinoma, and melanoma [130]. Of note, five of these PRNP gene somatic mutations were considered to be pathogenic since they affect PrP^C^ function, while four were identified as amyloid-prone PRNP somatic mutations [130]. Moreover, in pancreatic ductal adenocarcinoma cell line BxPC-3, Yang et al. [131] identified six missense mutations in four major genes involved in the GPI anchor modification pathway which contributes to increased tumor cell motility.

The multi-faceted role of PrP^C^ in tumorigenesis is greatly increased by recent findings showing how PrP^C^ in its folded conformation may foster tumor progression by regulating CSC oncogenic properties, such as self-renewal, pluripotency gene expression, and differentiation [21,23,132]. Remarkably, PrP^C^ over-expression has been related to the acquisition of a malignant phenotype of CSCs in a variety of solid tumors. In fact, high levels of PrP^C^ correlate with enhanced migratory and invasive capacities of CSCs [22,28,35,48], as well as increased resistance to anti-cancer agents [29,133,134]. Conversely, PrP^C^ depression impairs CSCs proliferation, migration, and invasive potential [22,29,32,50,51], while restoring cell sensitivity to chemo- and radiotherapy [56,135].

In the next section, we discuss evidence on the role of PrP^C^ as the main regulator of CSCs’ phenotype, biology, and functioning.

### 3.1. The Role of PrP^C^ in CSC Stemness and Differentiation

One of the first indications concerning the role of PrP^C^ in CSC biology dates back to the studies by Fan et al. [25,126], which provided strong evidence that over-expression of PrP^C^ at both protein and mRNA levels occurs in gastric carcinomas and gastric cancer cell lines and correlates with increased invasive potential and therapeutic resistance.

Later on, several lines of evidence propose that high expression of PrP^C^ contributes to cancer cell stemness and differentiation, which in turn became a hot topic in cancer research. For instance, Corsaro and colleagues (2016) provided evidence about a relevant role of PrP^C^ in conferring distinct stem-like features to human GBM CSCs, such as self-renewal ability and pluripotency [47]. Often referred to as glioblastoma stem-like cells (GSCs), these cells display up-regulated levels of PrP^C^ along with a higher expression of several stem cell markers including CD15, CD133, Nanog, Musashi-1, and Sox2 [20,22,47]. Remarkably, PrP^C^ is co-expressed and co-localizes with CD133 on the plasma membrane of neurospheres enriched in stem-like cells, suggesting that these molecules act as a functional complex to sustain GSC proliferation and stemness, while restraining them from differentiation. Conversely, CD133 expression was markedly decreased in PrP^C^-depleted neurospheres. Again, PrP^C^ down-regulation correlates with a marked suppression of stemness markers in GSCs, which in turn acquire a more differentiated, and thus less oncogenic, phenotype [47]. In fact, silencing PrP^C^ results in the up-regulation of cell differentiation markers, while abrogating GSCs self-renewal and neurosphere formation [22,47]. These data are in line with the evidence that PrP^C^ is key to maintain cancer stemness during colorectal cancer progression [125,133]. In fact, in human colorectal CSCs (CCSCs), PrP^C^ regulates the expression of various stem cell markers (i.e., Nanog, Sox2, ALDH1A1) and particularly Oct4, which governs CSC self-renewal and pluripotency [136]. On the other hand, PRNP knockdown markedly decreases stem cell marker expression as well as sphere formation ability in human CCSCs [125,133]. Notably, high levels of PrP^C^ expression identify a functionally distinct subpopulation of CD44-positive CCSCs which displays greater tumor-initiating and metastatic potential than PrP^C^-negative ones [48].

Collectively these data suggest that the presence of PrP^C^ is critical to maintain CSC stemness and that its reduction could represent a strategy to force CSC to shift towards a more differentiated and chemotherapy-sensitive phenotype.

### 3.2. The Role of PrP^C^ in CSC Growth and Proliferation

Recently, CSCs were reported to be highly dependent on PrP^C^ to maintain their growth and proliferation [49,125,128]. In fact, PrP^C^ promotes glucose uptake through Fyn-hypoxia-inducible factor (HIF)-2α-glucose transporter 1 (Glut1) signaling thus supporting CSC growth and survival [137].

Furthermore, consistent evidence indicates that PrP^C^ expression correlates with in vitro proliferation rate and in vivo tumor-initiating activity of CSCs, whereas silencing PrP^C^ strongly affects cell growth and clonogenic and tumorigenic potential [47,48]. For instance, in gastric cancer cells, PrPc up-regulation promotes CSC proliferation through PI3K/Akt activation and subsequent transcriptional activation of CyclinD1 to accelerate the G1/S phase transition [49]. Notably, pioneer studies revealed that endogenous PrP^C^ production in human GBM cell lines is dependent on the cell cycle phase, with the highest expression during the G1-phase [127]. This, in turn, indicates that PrP^C^ contributes to GSCs’ growth through G1/S phase transition and thus enhanced protein synthesis.

Again, PrP^C^ expression correlates with increased cell proliferation and tumorigenesis of human PDAC cell lines by potentiating Notch1 signaling [35]. Remarkably, these effects are reverted by PrP^C^ silencing through Notch1 down-regulation, and combining PrP^C^ and Notch1 inhibition is more effective than targeting single pathways alone. From a mechanistic point of view, PrP^C^ was shown to complex with Notch1 and to enhance Notch1 stability, thereby impairing proteasome-dependent Notch1 degradation. It is remarkable that down-regulation of Notch1, which occurs as a downstream effect of PrP^C^ silencing [35], inhibits the PI3K/Akt/mTOR pathway to abolish CSC stemness, self-renewal, invasiveness, and in vivo tumor growth in GBM [84]. Notably, these effects are reproduced by the ATG inducers AZD8055 and rapamycin, which suppress GSC self-renewal and abolish GSC tumorigenicity through degradation and inhibition of Notch1 [84,138].

Interestingly, most PrP^C^ functions appear to be linked to its binding partners [93]. For instance, PrP^C^-STI1/HOP binding induces GSC proliferation through the activation of PI3K/Akt and MAPK/ERK1/2 pathways [22,84,128]. Conversely, blockage of this interaction through a HOP peptide mimicking the PrP^C^-binding site (HOP_230–245_) impairs PI3K/Akt and Erk1/2 activation, thus affecting GCS proliferation [23]. Again, these effects are replicated in vivo where PrP^C^/HOP silencing has profound effects on tumor growth and animals’ survival. In fact, administration of HOP_230–245_ peptide to mice bearing GBM xenografts impairs in vivo tumorigenic potential of GSCs. This, in turn, results in a decreased tumor volume, while extending mice survival [23].

Collectively these data are in line with growing literature indicating that PrP^C^, either alone or through its binding partners, influences CSC stem cell characteristics, while affecting their growth, proliferation, and clonogenic ability. For example, in melanoma cells, the normal PrP^C^ exists as a pro-PrP. This latter represents a PrP isoform retaining its GPI anchor peptide signal sequence (GPI-PSS) that contains an FLNa binding motif and binds FLNa. The engagement of pro-PrP with FLNa facilitates the recruitment of integrin β1, which ultimately regulates cell proliferation, migration, and spreading, thus providing a growth advantage to melanoma cells [40]. In addition to FLNa, PrP^C^ interacts with Notch1, forming a PrP^C^/FLNa/Notch1 complex, which is associated with enhanced PDAC proliferation, invasiveness, and xenograft tumor growth [35,84]. Similarly, in gastric cancer cells, the interaction between PrP^C^ and the MGr1-Ag/37 kDa laminin receptor precursor protein (37LRP) is associated with a high tumor proliferation rate [26]. On the other hand, PrP^C^-induced proliferation in gastric cancer cells is significantly attenuated by inhibition of PI3K/Akt following the knockdown of the MGr1-Ag/37LPR binding partner [26].

### 3.3. The Role of PrP^C^ in CSC Migration and Invasion

Beyond its effects on CSC proliferation, stemness, and differentiation, it is also noteworthy that PrP^C^ over-expression correlates with CSC enhanced cell migration and invasion, and thus tumor-spreading and metastasis [35,40,41,126,139]. In fact, CSCs are reported to be highly dependent on PrP^C^ to maintain their increased invasive potential and metastatic abilities [32,35,48,126]. These, in turn, are among foremost key features of CSCs which enable them to disseminate within neighboring tissues and to metastasize to distant organs. Within this frame, several studies proposed that PrP^C^ is involved in the regulation of cell adhesion-related proteins [47,132,134,140]. This is the case of the stem cell marker CD44, a cell surface adhesion receptor involved in cell adhesion, motility, and metastasis [141]. In line with this, CD44-positive colorectal CSCs expressing PrP^C^ display enhanced disseminating capacity compared to PrP^C^-negative ones [48]. Remarkably, CD44 and PrP^C^ are co-expressed and co-localize on the cell membrane in human breast cancer cell line MCF7/Adr [132]. Activation of EGFR by binding with CD44/PrP^C^ results in increased CSC invasion and metastasis via up-regulation of cell adhesion-related proteins such as CD147, matrix metalloproteinase (MMP) 2 (MMP2), and MMP9 [132]. Conversely, siRNA-mediated PrPc depletion in MCF7/Adr cells significantly impairs CSC migration and invasiveness [47,132]. Notably, these effects are mimicked by monoclonal antibodies targeting PrP^C^ which selectively abrogates in vivo PrP^C^ pro-migratory functions, thereby restricting CSC to their primary tumor sites [48]. Similarly, RNAi-mediated PrP^C^ down-regulation inhibits both in vitro and in vivo CCSC tumorigenicity and invasiveness by abrogating epithelial to mesenchymal transition (EMT) related to ERK2 (MAPK1) pathway [48,84]. Consistently, higher PrP^C^ expression induces EMT in epithelial CRC cells through the modulation of E-cadherin and N-cadherin expression as well as β-catenin translocation from membrane to nucleus [139]. In schwannoma cells, PrP^C^ contributes to cell-matrix adhesion by activating the 37/67 kDa non-integrin laminin receptor (LR/37/67 kDa) and downstream FAK signaling pathway [21,84]. Additionally, PrP^C^ can bind to laminin, integrin, vitronectin, and/or HOP/STI1 to modulate CSC adhesion and metastasis formation [22,28,48,142].

Regarding lung adenocarcinoma, PrP^C^ is key to promote cancer cell lamellipodia formation, migration, and invasion via JNK signaling [37]. In fact, knockdown of PrP^C^ expression abrogates these in vitro effects, while decreasing in vivo experimental lung metastasis. This, in turn, is associated with reduced JNK phosphorylation and reduced protein levels of a transcriptional activator of the PRNP promoter, namely, the nuclear factor interleukin 3 (NFIL3) [37,84].

Furthermore, PrP^C^ up-regulation promotes the adhesive, invasive, and in vivo metastatic abilities of gastric cancer cells, thus strongly affecting gastric cancer malignant phenotype. This occurs, at least in part, through PrP^C^-dependent activation of the MEK/ERK pathway and MMP11 transactivation [126]. As a proof of concept, silencing PrP^C^ with siRNA produced a marked inhibition of in vitro invasive abilities in two gastric cancer cell lines, namely SGC7901 and MKN45 [126]. Similar results were reported in the human breast carcinoma cell line MCF7 [135]. In fact, PrP^C^ over-expression promotes breast CSC invasiveness through ERK and NF-kB-dependent activation of MMP9 promoter, whereas silencing of PrP^C^ inhibits breast cell migration and invasion [32]. Again, reducing PrP^C^ expression alters the spatial distribution of FLNa as well as actin filament organization, thereby impairing cell spreading and migration in human A7 melanoma cell lines [40]. In PDAC cell lines, PrP^C^ expression alters normal physiological functions of FLNa, thereby influencing in vitro CSC migration and invasiveness as well as in vivo tumor growth and infiltration [34].

Independently of its binding with FLNa, PrP^C^ affects the cytoskeletal organization by modulating the Akt-hsp27-F-actin axis, thus enhancing cancer cell migration in M2 melanoma cells which lack FLNa expression [41]. In particular, in FLNa-deficient M2 melanoma cells, PrP^C^ stabilizes Akt levels and its interaction with hsp27 to regulate hsp27 phosphorylation, actin polymerization, and thus cell migration [41].

#### 3.3.1. The Role of PrP^C^ in Tumor Spreading and Neuro-Invasion

Recently, it was suggested that PrP^C^ might be involved in tumor spreading along the perineural pathway, also known as perineural invasion (PNI), and thus distant metastasis.

This is the case, for instance, of colorectal, gastric, and prostate cancer cells that use PNI as a route for tissue dissemination and tumor-spreading to distant organs [143,144]. Although the association between PrP^C^ and PNI is yet poorly characterized, Zhou et al. (2014) report that in gastric cancer, PrP^C^ is associated with several clinic-pathological parameters including depth of invasion and lymph node metastasis [54]. In line with this, increased levels of PrP^C^-positive cells are identified in the lymph nodes that are invaded by colorectal CSC [145]. Moreover, high levels of PrP^C^ are present in colorectal CSC that are found invading tumor stroma and metastasis of liver and lung [145].

Again, extra-pancreatic PNI and nodal involvement along known peripancreatic neural plexuses occur in up to 100% of pancreatic cancers [36]. Being detected already at early stages, extra-pancreatic PNI negatively impact on the overall survival of PDAC patients [146]. Remarkably, preliminary data on surgically resected specimens of PDAC disclose a potential correlation between increased PrP^C^ expression and PNI [36]. Although not significant so far, these preliminary data are encouraging since they evidence a trend towards a higher PrP^C^ expression in PDAC patients with PNI [36]. Notably, the presence of PrP^C^ within PDAC tissue might be a marker that could contribute to explaining the biology of the disease in terms of aggressiveness, explaining the uniquely preferred PNI of PDAC, based on the relationship of prions with neurotropism and neurodegenerative disease [36,147]. In this scenario, an analogous significance for PrP^C^ in GBM has revealed how tumor diffusion is reminiscent of the spreading mechanisms in neurodegeneration. This implies a relationship between PrP^C^ and neurotropism, which again is a biological peculiarity of PDAC [36,84].

Even in the cases eligible for surgical resection, the poor prognosis of most of these solid tumors is due to an extensive local infiltration and to an early lymphatic spreading [36]. In fact, after surgical resection, most of these solid tumors frequently recur both within the primary site and/or to distant, secondary sites. Thus, this aspect deserves particular mention and advances in understanding the biology of tumor PNI are urgently needed.

#### 3.3.2. PrP^C^ Spreading and PrP^C^-Mediated Epigenetic Effects

Recent findings suggest that PrP^C^ can take part in cell-to-cell communication within the tumor microenvironment (TME) acting both as an autocrine and/or paracrine signaling molecule to foster CSC malignant phenotype. For instance, PrP^C^ is abundantly released from schwannoma cells, either as a free cleaved peptide or via exosomes, to promote malignant cell growth in an autocrine fashion [21]. In addition, when functioning in a paracrine mode, secreted PrP^C^ can disseminate within the TME to activate various downstream pathways (i.e., ERK1/2, PI3K/Akt, FAK) within neighboring cells, thus promoting tumor growth and infiltration [21,47]. In breast cancer, secreted PrP^C^ can directly sequester chemotherapeutic drugs, blocking their cytotoxic activity, thus providing a growth advantage to breast CSCs [84,148]. Conversely, genetic depletion of PrP^C^ prevents such an interaction, while sensitizing breast CSCs to chemotherapy [84,148].

In line with this, the PrP^C^ cell surface ligand STI1/HOP can be translocated to the cell surface and/or secreted outside the tumor cell, acting both as an autocrine and paracrine factor in promoting cell growth, proliferation, and migration of various tumor cells, encompassing ovarian [149,150], renal [151], and glioma (Erlich et al., 2007) [128] cancer cells. In the case of GBM, Erlich and colleagues (2007) report that STI1 is secreted by and induces tumor cell proliferation in human A172 GBM cell line through MAPK and PI3K pathways [128]. Moreover, Wang et al. (2017) demonstrate that STIP1/HOP promotes osteolytic bone metastasis in renal cell carcinoma (RCC) both in an autocrine and paracrine manner [151]. In particular, when exposed on the extracellular surface, STIP1 activates autocrine STIP1-ALK2-SMAD1/5 signaling in bone metastatic RCC tumor cells thus promoting cell proliferation, migration, and invasion [151]. In addition, STIP1 can be secreted within the extracellular space to activate the paracrine STIP1-PrP^C^-ERK1/2 pathway which promotes osteoclasts differentiation and aggravates osteolysis [151]. Importantly, this results in a vicious cycle between tumor cell and bone niche which contributes to the early osteolytic progression, a recurring feature of RCC bone metastasis [151,152]. Notably, treatment with anti-STIP1 and/or anti-PrP^C^ antibody significantly abrogates these effects in both enriched bone-seeking RCC cell line OS-RC-2-BM5 and the murine preosteoclast cell line RAW264 [151].

Finally, it is worth of mentioning that emerging evidence suggests that PrP^C^ can act as an epigenetic modulator of tumor invasiveness and metastatic potential. Remarkably, Wang et al. (2012) show that PrP^C^ mediates colorectal cancer cell invasive and metastatic capacities by regulating SATB1 expression via epigenetic activation of the Fyn-SP1 pathway [139]. In particular, the authors demonstrate direct binding of SP1 to the SATB1 promoter, which is largely abolished by PrP^C^ depletion [139]. On the other hand, PrP^C^ overexpression accelerates tumor metastasis by upregulating SATB1 expression. This latter, which is a matrix attachment region (MARAR)-binding protein, is a master regulator of global gene expression conferring an aggressive, pro-metastatic phenotype in colorectal cancer cells [139].

### 3.4. Role of PrP^C^ in Multidrug Resistance (MDR)

Compelling evidence support the view that PrP^C^ may mediate protection of CSC from apoptosis, along with the acquisition of multidrug resistance (MDR), which in turn are responsible of treatment failure, tumor recurrence, and poor survival of cancer patients [30,48]. Remarkably, high PrP^C^ expression correlates with MDR in CSCs as shown in gastric, breast, glioma, and colorectal cancers [25,29,31,49,126,153]. For instance, in gastric carcinoma, PrP^C^ over-expression provides gastric cancer cells with increased resistance to doxorubicin [126]. In the case of GBM, PrP^C^ has shown to exert a cytoprotective role in different glioma cell lines, conferring resistance to apoptotic cell death [154]. Similarly, Meslin et al., (2007) [31] demonstrate that PrP^C^ over-expression in breast cancer cells is associated with resistance to tumor necrosis factor-related apoptosis-inducing ligand (TRAIL)-induced apoptosis. Conversely, down-regulation of PrP^C^ by siRNA sensitizes breast cancer cells to adriamycin and TRAIL-mediated cell death [31].

In line with this, PrP^C^ suppresses adriamycin-induced apoptosis and alters Bcl-2 and Bax expression, which may be another pathway contributing to PrP^C^-related MDR in SGC7901/ADR gastric cancer cell lines [25]. In particular, in these tumor cells, PrP^C^ confers resistance of both P-glycoprotein (P-gp)-related and P-gp-non related drugs, which is accompanied by decreased accumulation and increased releasing amount of adriamycin in PrP^C^-overexpressing cells. Interestingly, Pan and colleagues (2005) [25] demonstrate that PrP^C^ upregulates the expression of P-gp, an ATP-binding cassette (ABC) drug efflux pump, thus allowing the cell to evade cytotoxic attacks from a variety of chemotherapeutic agents via its anti-apoptotic activity. Similarly, in breast cancer, PrP^C^ physically interacts and co-localizes with P-gp on the cell membrane of MDR breast cancer cell line MCF7/Adr [135]. Disruption of PrP^C^/P-gp complex has profound effects on tumor survival and aggressiveness, since it markedly reduces the anti-apoptotic activity, while promoting the reversal of PrP^C^-mediated drug resistance in MDR breast cancer cells [135]. Furthermore, the direct interaction between PrP^C^ and EGFR is determinant for cisplatin/oxaliplatin resistance in colorectal cancer cells via FOXO3a-Krüppel-like factor 5 (KLF5) signaling, thus contributing to the development of metastases and poor outcome in CRC patients [155]. Again, PrP^C^/CD44 interaction promotes chemoresistance and tumor progression in MDR breast cancer cells [132].

It is important to remark that several beneficial effects including sensitization to chemotherapy are obtained by means of PrP^C^-targeted strategies resulting in PrP^C^ inhibition/downregulation. In fact, both anti-PrP^C^ antibodies and/or silencing PrP^C^ expression by antisense of RNAi technology were shown to reverse, at least in part, the MDR phenotype of CSC in various solid tumors [25,51,84,126,132].

## 4. PrP^C^ as a Potential Biomarker in Human Cancers

Beyond its effects on cancer cell biology and function, compelling evidence suggests that PrP^C^ expression rate may be of clinical relevance in several malignancies. This issue is of particular interest in cancer research since it may provide early detection of high-risk pre-malignant and early-stage cancerous lesions, which in turn would facilitate early diagnosis and therapeutic intervention. For instance, Le Corre and colleagues (2019) [45] reported that plasma levels of PrP^C^ are elevated in metastatic CRC patients compared with healthy subjects, thus suggesting that PrP^C^ may serve as a potential biomarker for patient stratification in CRC [45]. Furthermore, high levels of circulating PrP^C^ in metastatic CRC patients’ plasma is predictive of poor prognosis [45]. In line with this, high-resolution cell surface proteomic evaluation identified PrP^C^ as a potential biomarker for colorectal adenoma-to-carcinoma progression, which could potentially discriminate normal colon and low-risk adenomas from high-risk adenoma and early-stage colorectal cancer patients [53].

**Figure 2:**
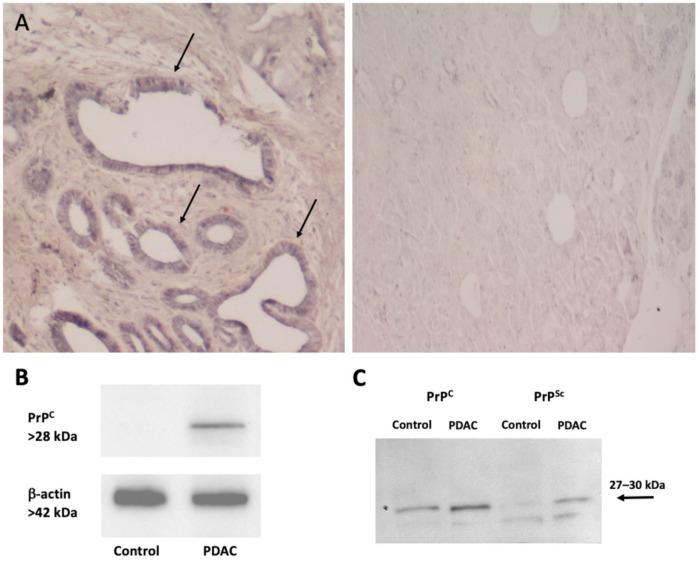
PrP^C^ expression in human pancreatic tissues. (**A**) The figure reports representative pictures of PrP^C^ immunohistochemistry of pancreatic ductal adenocarcinoma (PDAC) tissue (left panel) compared with control tissue (right panel). Immunoperoxidase shows PrP^C^-specific labelling in ductal cells (arrows) from a human tumor sample (left panel). Remarkably, the increase in PrPc expression occurs along with a marked loss of cellular architecture within pancreatic tumoral tissue, which features enlarged and irregularly shaped ducts within abundant extracellular matrix. In contrast, normal human pancreas (right panel) possesses a well-preserved architecture of both acinar cells and ductal system, along with a weak PrP^C^-staining in the ductal epithelial cells (original magnification 10×). (**B**) The figure reports a representative immunoblot for PrP^C^ and the house keeping protein β-actin in control and PDAC tissues. (**C**) The figure reports representative western blotting comparing scrapie prion protein (PrP^Sc^) and PrP^C^ (with or without proteinase K) in control and PDAC tissues as measured in Table 1. Images were obtained by an author of the manuscript (M.A. Giambelluca). Original western blot images (Figures S1 and S2) and densitometry readings (Tables S1 and S2) were provided in Supplemental Materials.

Notably, numerous lines of evidence support the potential role of PrP^C^ in regulating cancer sensitivity to chemotherapy, and thus monitoring therapeutic efficacy and patients’ prognosis. For instance, Wang JH et al. (2011) [55] reported that a high PrP^C^ expression in gastric cancer patients predicts both a poorer response to chemotherapy and a poorer prognosis. In fact, when compared with low PrP^C^ expression group, patients with higher PrP^C^ expression show increased resistance to chemotherapy, a lower 2-year survival rate and higher mortality rate [55]. Accordingly, PrP^C^ over-expression in estrogen receptor (ER)-negative breast cancer patients is associated with a lower sensitivity to chemotherapy, thus suggesting that PrP^C^ could be predictive for the benefit of adjuvant chemotherapy in ER-negative patients [56]. Expression of PrPc in human PDAC biopsies is associated with a worse survival than PrP-negative cases, supporting a critical tumor-promoting role of PrP^C^ in PDAC [35]. Remarkably, western blot and immunohistochemical analyses from surgically resected specimens of PDAC demonstrate a marked difference between PDAC tissues compared to control. In detail, in PDAC specimens PrP^C^ is overexpressed very selectively within the ductal compartment, whereas normal control tissues feature only few ductal epithelial cells with moderate PrP-staining [36] (Figure 2). Notably, PrP^C^ expression was independent of the presence of dysplastic areas surrounding “healthy” pancreas, thus indicating that PrP^C^ levels correlates with tumor invasiveness and aggressiveness, and not with preneoplastic lesions [36]. Although the mean patients’ follow-up is yet too short to correlate PrP^C^ expression to clinical outcome, the authors report a significant difference between groups when analyzing the possible relationship between PrP^C^ and cancer stage after resection based on pTNM [36]. Even if these data are yet preliminary in identifying PrP^C^ as a potential biomarker in the follow-up of patients following surgical resection, they are nevertheless encouraging. However, further studies are needed to completely define a prognostic value of PrP^C^ detection.

In addition, as reported by Du et al. (2005) [25], PrP^C^ is ubiquitously expressed in gastric carcinoma cell lines and tissue, while negatively or weakly expressed in normal gastric mucosa and adjacent non-tumoral tissues. Remarkably, PrP^C^ overexpression correlates with histopathological degree of differentiation and disease progression in gastric adenocarcinomas, being significantly higher in poorly-differentiated gastric carcinoma [25,54]. In line with this, pioneer studies in primary brain tumors identified endogenous PrP^C^ as being highly expressed within human GBM cell lines constitutively [127,156]. Similarly, Comincini and colleagues [20] found that GBM tumor samples display overexpression in PrP^C^ and prion-like protein Doppel, whilst PRND expression directly relates to tumor malignancy, thus being associated with a worse prognosis in high-grade astrocytoma [84]. Recently, an association between PrP^C^/STI-1(HOP) expression and lower patient survival has been confirmed in human astrocytoma samples. In detail, human GBM samples feature an increased expression in PrP^C^/STI-1(HOP) compared with low-grade astrocytoma (grade I–III) or non-tumoral tissue [23,84]. Again, higher PrP^C^-positive rates and stronger PrP^C^ staining are found in breast [56], colorectal [42], gastric cancer [54,55], and head and neck squamous cell carcinoma (HNSCCs) [38]. Although the PrP^C^-positive rates may vary between different cancer cell types, collectively these data demonstrate that increased PrP^C^ expression closely associates with the pathological degree and clinical progression of human cancers, while emphasizing a common molecular mechanism underlying the contributory role of PrP^C^ in tumor progression. However, further studies with larger sample sizes and in different cohorts of patients are needed in order to completely define the diagnostic and prognostic value of PrP^C^ detection. Nonetheless, it is fascinating that PrP^C^ emerges as a kernel within a network to link the CNS to peripheral organs even in physiological conditions, which is paradoxically occurring in cancer.

## Figures and Tables

**Table 1:** PrP^C^ compared with PrP^Sc^ in control and PDAC tissues.

Protein	Control	Tumor	Ratio T/C
PrP^C^	32.80 ± 3.5	44.34 ± 5.4	1.35 ± 0.1
PrP^Sc^	22.57 ± 4.1	50.36 ± 6.8 *	2.26 ± 0.1
Ratio PrP^Sc^/PrP^C^	0.68 ± 0.1	1.13 ± 0.1 *	1.70 ± 0.1

Western blotting with (PrP^Sc^) or without (PrP^C^) pre-treatment with proteinase K in control and PDAC tissues were compared and their ratio was provided. In PDAC tumor tissue, the increase in PrP^Sc^ surpasses at large that reported for PrP^C^. Values are given as the mean ± S.E.M. optical density * *p* < 0.05 compared with control.

## Supplementary Materials

The following are available online at https://www.mdpi.com/2072-6694/13/2/170/s1.
