# Supplementary material for: The Role of Cellular Prion Protein in Promoting Stemness and Differentiation in Cancer
Source: Cancers (Basel). 2021 Jan 6;13(2):170. doi: 10.3390/cancers13020170 (PMC7825291; doi:10.3390/cancers13020170)
Supplement: Supplementary file 1 [file cancers-13-00170-s001.pdf]

Supplementary Materials: The Role of Cellular Prion Protein in Promoting Stemness and Differentiation in Cancer

Larisa Ryskalin, Francesca Biagioni, Carla L. Busceti, Maria A. Giambelluca, Luca Morelli, Alessandro Frati and Francesco Fornai

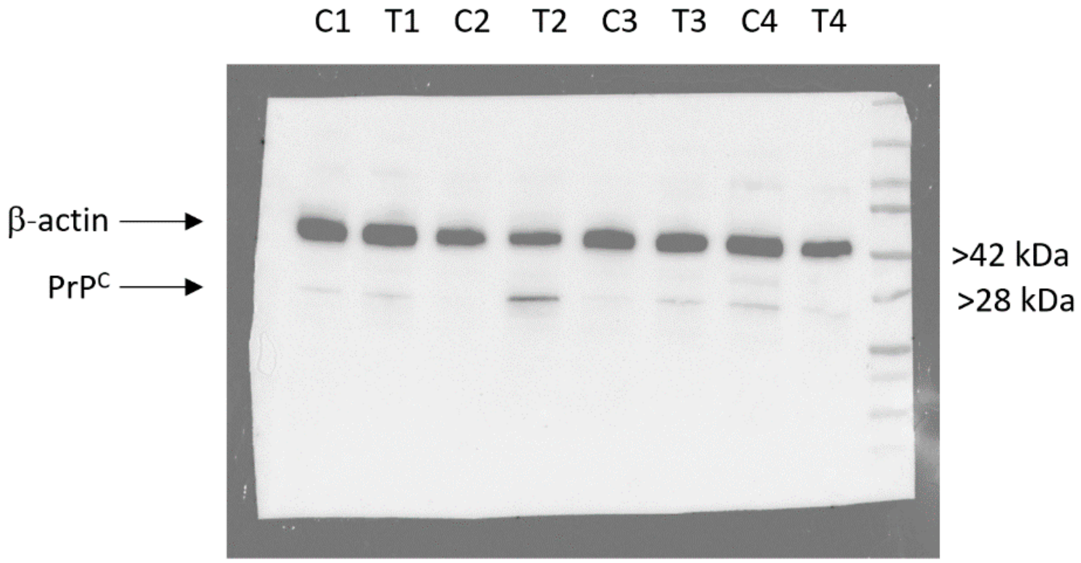

Figure 1. Original western blot image of Figure 2B, reporting immunoblot for PrP^C and the house keeping protein β–actin in control (C) and PDAC (T) tissues from different surgically resected specimens of PDAC.

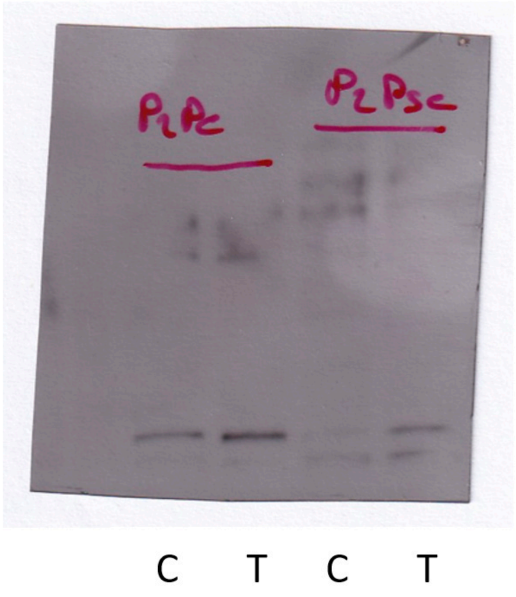

Figure 2. Original western blot image of Figure 2C, comparing scrapie prion protein (PrP^{Sc}) and PrP^C (with or without proteinase K) in control (C) and PDAC (T) tissues.

Table 1. Densitometric analysis of PrP^C expression in control (C) and PDAC (T) tissues.

Sample	PrP^C	β-actin	PrP^C/β-actin
C 1	932.65	31,773.81	0.029
T 1	1911.91	32,883.96	0.058
C 2	68.95	24,268.30	0.003
T 2	11,492.14	21,705.64	0.529
C 3	790.34	27,486.59	0.029
T 3	1898.03	26,837.94	0.071
C 4	2963.18	29,874.35	0.099
T 4	860.75	22,088.16	0.039

Table 2. Densitometric analysis of PrP^C and PrP^{Sc} expression in control (C) and PDAC (T) tissues.

Protein	Control	Tumor	Ratio T/C
PrP^C	34.860	42.270	1.212
PrP^{Sc}	19.800	44.833	2.264
